# Low-Grade Myxofibrosarcoma of the Rectus Abdominus Muscle Infiltrating into Abdominal Cavity: A Case Report

**Published:** 2017-02-21

**Authors:** Tadashi Nomura, Shunsuke Sakakibara, Aya Moriwaki, Teruya Kawamoto, Satoshi Suzuki, Takeshi Ishimura, Kazunobu Hashikawa, Hiroto Terashi

**Affiliations:** ^a^Department of Plastic Surgery, Kobe University Graduate School of Medicine, Kobe, Hyogo, Japan; ^b^Department of Orthopaedic Surgery, Kobe University Graduate School of Medicine, Kobe, Hyogo, Japan; ^c^Division of Gastrointestinal Surgery, Department of Surgery, Kobe University Graduate School of Medicine, Kobe, Hyogo, Japan; ^d^Division of Urology, Department of Surgery Related, Kobe University Graduate School of Medicine, Kobe, Hyogo, Japan

**Keywords:** abdominal cavity, myxofibrosarcoma, rectus abdominis muscle

## Abstract

**Objective:** Myxofibrosarcoma (MFS) is a relatively rare tumor that is histologically characterized by myxoid stroma and spindle cell proliferation. This tumor most commonly arises as a slow growing, enlarging painless mass in the extremities of elderly patients. **Methods:** We report a case of a primary, low-grade MFS in the rectus abdominis muscle infiltrating the abdominal cavity of a 75-year-old man. **Results:** The patient underwent a wide excision of the right abdominal wall mass with a 3-cm surgical margin from the scar due to a biopsy. The tumor infiltrated the urinary bladder, peritoneum, and external iliac vessels. Twenty-six months after the initial operation, he had recurrences in his abdominal wall, urinary bladder, and right iliac vessels. **Conclusions:** To our knowledge, primary MFS of the muscle in the abdomen has not been documented previously. Although this case was histopathologically classified as a low-grade tumor, it infiltrated the abdominal cavity. The tumor is suspected to have penetrated the abdominal cavity below the linea arcuata, which lacks the posterior sheath of the rectus abdominis muscle; from there, it could easily spread without being blocked by any biological barriers.

Myxofibrosarcoma (MFS) is a relatively rare tumor that is histologically characterized by myxoid stroma and spindle cell proliferation. This tumor most commonly arises as a slowly enlarging painless mass in the extremities of elderly patients and is very rarely observed in the abdominal cavity and retroperitoneum.^1^To our knowledge, primary MFS of the muscles in the abdomen has not been previously documented. We report a case of a primary low-grade MFS in the rectus abdominis muscle invading the abdominal cavity of a 75-year-old man.

## METHODS

**Figure 1:**
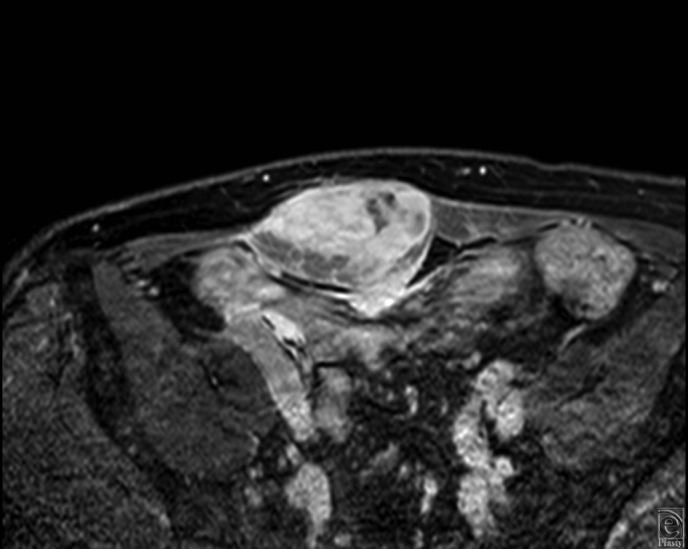

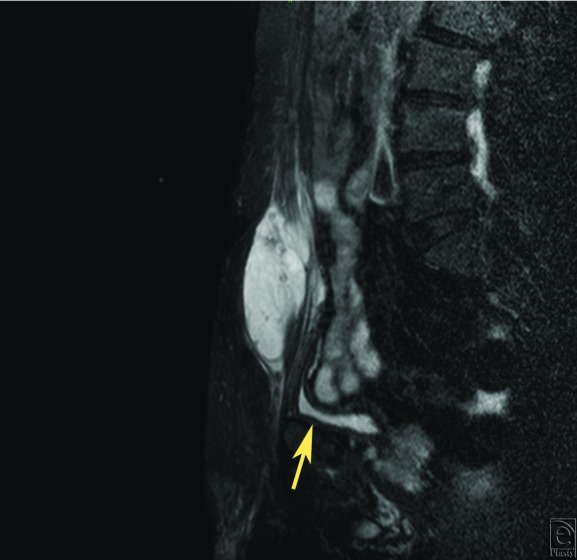
(a). Axial section of gadolinium-enhanced T_1_-weighted magnetic resonance imaging. The gross tumor was located in the right rectus abdominis muscle and spread horizontally. (b). Sagittal section of T_2_-weighted magnetic resonance imaging. The abdominal tumor did not spread into the abdominal cavity. The yellow arrow indicates the urinary bladder.

A 75-year-old man experienced an induration with pain on his lower abdominal wall. Since the tumor increased gradually, he consulted a plastic surgeon. Magnetic resonance imaging revealed a tumor in his right rectus abdominis muscle. Magnetic resonance imaging showed a heterogeneously contrast-enhancing mass involving his right rectus abdominis muscle (Fig 1). The patient underwent a biopsy at the Department of Orthopaedic Surgery in our hospital. Low-grade MFS was diagnosed histopathologically and the patient was referred to our department for further management including reconstructive surgery. No other lesions were detected on whole-body fluorodeoxyglucose-positron emission tomography (PET) scanning. At this point, there was no apparent evidence whether the tumor invaded into the abdominal cavity or not.

**Figure 2:**
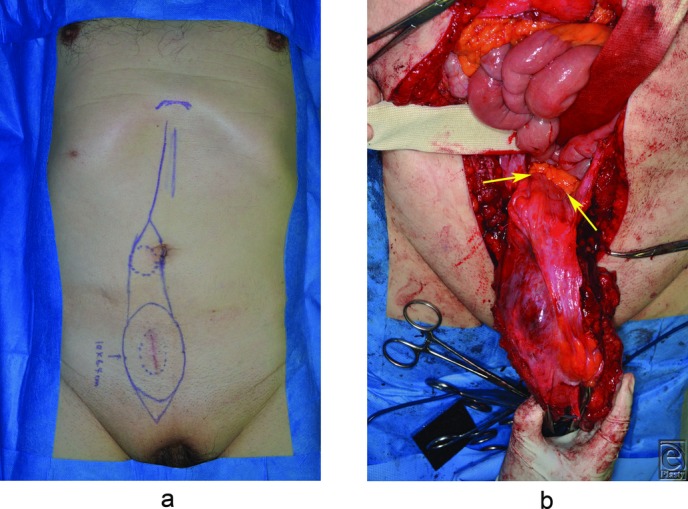
Intraoperative view. (a). Skin incision line. (b). The tumor invading the abdominal cavity (arrow).

The patient underwent a wide excision of the right abdominal wall mass with a 3-cm surgical margin from the scar due to a biopsy. The tumor infiltrated the urinary bladder, so we shaved the surface of the muscle layer of the bladder (Fig 2). However, the firm and fibrous mass was infiltrating the peritoneum and external iliac vessels. As the margin was described as positive or involved in intraoperative rapid diagnosis, further excision was performed. Consequently, the entire right rectus abdominis muscle along with the overlaying skin and the subcutaneous tissue, part of the bladder muscle, the right half of the posterior peritoneum, and the adventitia of the external iliac vessels. We attached Seprafilm (Kaken Pharmaceutical Co, Ltd, Tokyo, Japan) to the defect of the posterior peritoneum. We reconstructed the abdominal wall with Composix E/X Mesh (Davol, A Bard Company, Rhode Island).

## RESULTS

**Figure 3:**
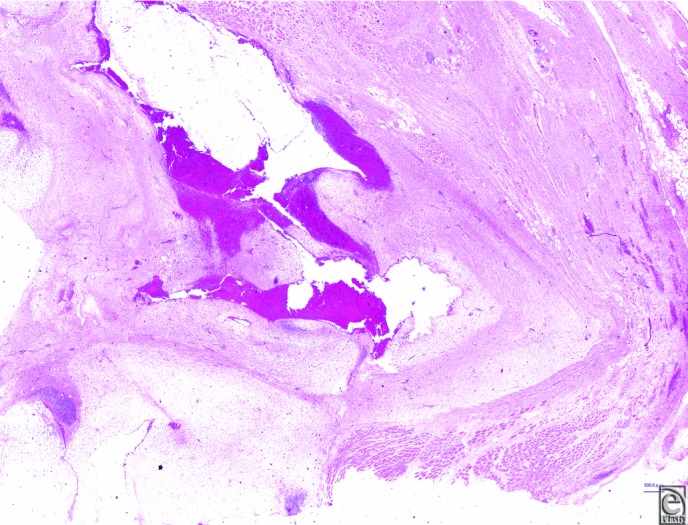

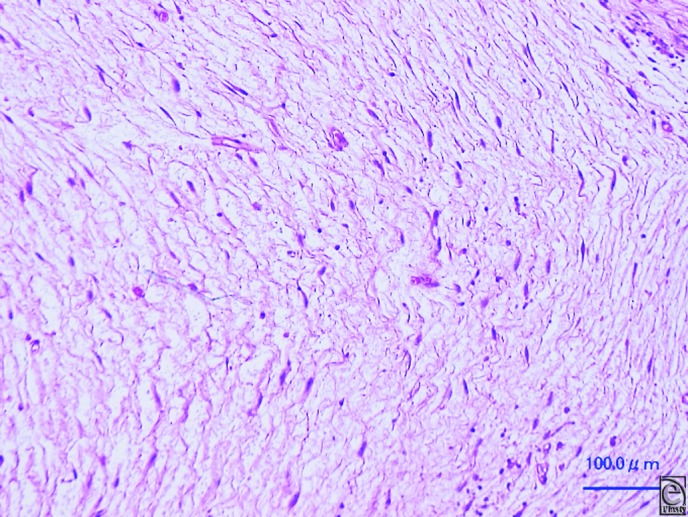
Histological findings (hematoxylin and eosin staining). (a). Low-power view of the lesion showing infiltrative growth into the muscle layer. The internal part of it had hemorrhage (scale bar indicates 500 μm). (b). Higher power magnification showing spindle, stellate, and pleomorphic cells (scale bar indicates 100 μm).

Postoperatively, the patient had a paralytic ileus, which resolved after decompression with an ileus tube. The gross specimen measured 10 × 4 × 4 cm, and the lesion within the specimen was semitranslucent, gelatin-like, and with hemorrhage. Histologic examination showed a multinodular lesion with a prominent myxoid matrix. A small number of irregular, spindle-shaped, or stellate cells, with hyperchromatic nuclei, proliferated in the stroma (Fig 3). According to the modified French Fédération Nationale des Centres de Lutte Contre le Cancer (FNCLCC) grading system, the tumor in this cases scored 3 (tumor differentiation; score 2, mitotic count; score 1, tumor necrosis; score 0), that is, grade 1. Twenty-six months after the initial operation, a recurrence was prominent in his right abdominal wall, urinary bladder, and the right iliac vessels. Considering the patient's physical condition, it was very difficult for our multidisciplinary medical team to come to a consensus regarding ablative surgery including radical cystectomy and removal of the right external iliac vessels. During chemotherapy with doxorubicin, he complained of nausea and fatigue. At that point, he opted for the best supportive care without further medical intervention.

## DISCUSSION

Myxofibrosarcoma is one of the most common soft tissue sarcomas in the elderly and occurs mostly on extremities.^1^ It was first described in 1977 as a distinct soft tissue sarcoma characterized by a mucoid and nodular appearance.^2^ Most cases of this tumor occur in the dermis or the subcutaneous tissue.^3^ It usually presents as a painless, slow-growing mass. Currently, the World Health Organization classifies MFS as a fibroblastic/myofibroblastic malignant tumor with specific clinicopathological characteristics. Histopathological features show multinodular growth with incomplete fibrous septa and a myxoid stroma composed of hyaluronic acid.^4^ In low-grade lesions, mitotic figures are infrequent.^4^^,^^5^ The present case had a multinodular lesion with a prominent myxoid matrix and slightly increased mitotic activity on light microscopic examination. A small number of irregular, spindle-shaped, or stellate cells, with hyperchromatic nuclei, proliferated in the stroma. This case was diagnosed as a malignant soft tissue tumor, MFS. In FNCLCC grading system, the tumor scored 3 (tumor differentiation; score 2, mitotic count; score 1, tumor necrosis; score 0), that was grade 1 (low-grade) disease.

In the present case, this large tumor might have originally been located in the rectus abdominis muscle. It extended into the abdominal cavity, involving the urinary bladder and the right half side of the peritoneum. To our knowledge, MFS in the skeletal muscle of the trunk is rare, with few cases of thoracic lesions being reported previously.^6^^,^^7^ There are only 2 previously reported cases of MFS of the retroperitoneum from Japan.^8^^,^^9^ Moreover, there have been no reports in the English literature describing cases similar to our case, with infiltrative growth into the abdominal cavity. In this case, the tumor penetrated the abdominal cavity below the linea arcuata, which lacks the posterior sheath of rectus abdominis muscle; from there, the tumor could easily further infiltrate surrounding areas without being blocked by biological barriers.

Potential therapeutic options for MFS have been reported, including wide local excision, chemotherapy, and radiation. Wide local excision is the most recommended strategy for low-grade MFS.^7^ The local recurrence rate in patients with MFS after resection is 16% to 54%.^10^ Patients with close surgical margins should be considered for radiation therapy, and recurring masses should be widely excised, including the scar from the previous surgery.^11^ In the present case, owing to the wide defect of the abdominal wall after wide resection, we had to reconstruct the abdominal wall, using artificial mesh material. Despite the possibility of an inadequate excision, we could not administer postoperative radiation in order to prevent complications, such as an adhesive intestinal obstruction or exposure of the mesh. Chemotherapy with doxorubicin had been suspended because of side effects. Consequently, we could not perform curative treatment for this recurrent mass. Palliative therapy to increase the patient's comfort was important.

## CONCLUSION

We presented a rare case of a large tumor with clinical and histopathological features of low-grade MFS in the rectus abdominis muscle with recurrence. The tumor was suspected to have penetrated the abdominal cavity below the linea arcuata, which lacks the posterior sheath of the rectus abdominis muscle; from there, the tumor could easily infiltrate the surrounding areas without being blocked by biological barriers.

## References

[1] Goldblum JR, Flope AL, Weiss SW, Goldblum JR, Flope AL, Weiss SW (2014). Borderline and malignant fibroblastic/myoblastic tumors. Soft tissue tumors.

[2] Angervall L, Kindblom LG, Merck C (1977). Myxofibrosarcoma. A study of 30 cases. Acta Pathol Microbiol Scand A.

[3] Mentzel T, Calonje E, Wadden C (1996). Myxofibrosarcoma. Clinicopathologic analysis of 75 cases with emphasis on the low-grade variant. Am J Surg Pathol.

[4] Mentzel T, van den Berg E, Molenaar WM, Fletcher CDM, Unni KK, Mertens F (2002). Myxofibrosarcoma. World Health Organization Classification of Tumours. Pathology and Genetics of Tumours of Soft Tissue and Bone.

[5] Scoccianti G, Ranucci V, Frenos F (2016). Soft tissue myxofibrosarcoma: a clinico-pathological analysis of a series of 75 patients with emphasis on the epithelioid variant. J Surg Oncol.

[6] Gopalratnam K, Rodriguez JA, Woodson KA, Folman R (2016). A case of myxofibrosarcoma in an unusual thoracic location. Case Rep Oncol.

[7] McMillan RR, Sima CS, Moraco NH (2013). Recurrence patterns after resection of soft tissue sarcomas of the chest wall. Ann Thorac Surg.

[8] Shimomura A, Hashimoto M, Moriyama J (2012). Long-term survival of a case of myxofibrosarcoma with 4 episodes of recurrence and resection. Jpn J Gastroenterol Surg.

[9] Nakamura K, Kitagami H, Hayakawa T (2013). A case of myxofibrosarcoma of the retroperitoneum. Jpn J Gastroenterol Surg.

[10] Hambleton C, Noureldine S, Gill F (2012). Myxofibrosarcoma with metastasis to the lungs, pleura, and mediastinum: a case report and review of literature. Int J Clin Exp Med.

[11] Mutter RW, Singer S, Zhang Z (2012). The enigma of myxofibrosarcoma of the extremity. Cancer.

